# Analysis on the Meiosis-Related Gene (Dmc1, Ph1) Expression in Autotriploid *Carassius auratus*

**DOI:** 10.1007/s10126-019-09921-x

**Published:** 2019-09-14

**Authors:** Qinbo Qin, Yuwei Zhou, Chongqing Wang, Minghe Zhang, Huan Qin, Chun Zhao, Shaojun Liu

**Affiliations:** State Key Laboratory of Developmental Biology of Freshwater Fish, College of Life Sciences, Hunan Normal University, Changsha, 410081 Hunan People’s Republic of China

**Keywords:** Triploid, Meiosis, Meiosis-related gene, Methylation, Expression

## Abstract

Triploid is usually considered to be unable to perform normal meiosis due to the abnormal behavior of the three sets of chromosomes. But autotriploid *Carassius auratus* in the Dongting water system (3n = 150, abbreviated as 3nCC) can perform normal meiosis. In artificial autotriploid *Carassius auratus* (3n = 150, abbreviated as 3nRR), female individuals undergo normal meiosis and produce mature gametes, while male individuals cannot. To better understand the effects of triploidization on meiosis in fish, we study the structure, methylation level, and expression level of meiosis-related genes (*Dmc1*, *Ph1*) in diploid *Carassius auratus* (2n = 100, abbreviated as 2nCC), *Carassius auratus* red var.(2n = 100, abbreviated as RCC), 3nCC and 3nRR. The results show that, compared with their diploid ancestors (2nCC and RCC), *Dmc1* and *Ph1* genes are hypomethylated in all 3nCC and female 3nRR, while are hypermethylated in male 3nRR. Correspondingly, *Dmc1* and *Ph1* genes are highly expressed in all 3nCC and female 3nRR, while are lowly expressed in male 3nRR. These results indicate that high expression of meiosis-related genes can contribute to restoration of bivalent pairing during meiosis in autotriploid *Carassius auratus*. This study provides new insights into the effect of DNA methylation on the fertility in triploid fish.

## Introduction

Polyploidization, the addition of a complete set of chromosomes to the genome, represents one of the most dramatic mutations known to occur (Mallet 2007; Otto 2007). Interspecific hybridization normally results in genome-level alterations, including the occurrence of triploid and tetraploids (Liu and Wendel 2003; Liu et al. 2007). Polyploids have been classified into autopolyploids and allopolyploids (Comai 2005; Otto 2007). Autopolyploids exhibit multivalent pairing during meiosis, in which an additional set (or sets) of chromosomes may originate from the same species (Qin et al. 2019). However, allopolyploids result from the combination of sets of chromosomes from different species, which undergo bivalent pairing at meiosis because of only a homologous chromosome pair (Qin et al. 2014b).

Meiosis is the fundamental process by which the gametes are formed in all sexual organisms (Zickler and Kleckner 2015; Bishop et al. 2017). *Dmc1* (DNA meiotic recombinase 1) and *Ph1* (polyhomeotic-like protein 1) as important meiosis-related genes control the behavior of chromosomes during meiosis. The expression product of *Dmc1* is a recombinant enzyme used in meiosis and plays a vital role during synapsis (Li and Ma 2006). DMC1 plays an important role in the exchange of DNA chains between homologous chromosomes and the repair of DSB (DNA double-strand breaks) during meiosis (Chen et al. 2016). Importantly, DMC1 deficiency causes the abnormal formation of synaptonemal complexes and disordered separation of homologous chromosomes (Kagawa and Kurumizaka 2010). In addition, *Ph1* locus is responsible for the specific induction of centromere association (Martinez-Perez et al. 2001). Correct pairing of homologous chromosomes is controlled by the *Ph1* that triggers a conformational change in chromatin by “perceiving” the degree of homology of the chromosome, thereby controlling chromosome pairing and recombination (Riley and Chapman 1958; Martinez-Perez and Moore 2008; Boden et al. 2010). Thus, *Ph1* gene allows homologous chromosome pairing to prevent partial homologous chromosome pairing in hybrids and polyploid (Al-Kaff et al. 2008).

In the Dongting water system, autotriploid form (3n = 150, abbreviated as 3nCC) was found in the *Carassius auratus* complex, in which normal gametes were produced during breeding season and the mean DNA content of sperm was half of that of somatic cell (Xiao et al. 2011; Qin et al. 2016). It provides direct evidences that 3nCC can perform normal bivalent pairing and then produce reduced gamete. In our previous study, we have artificially established an autotetraploid fish line (RRRR, 4n = 200, abbreviated as 4nRR) derived from the distant hybridization of *Carassius* auratus red var. (RR, 2n = 100, abbreviated as RCC) (♀) × *Megalobrama amblycephala* (BB, 2n = 48, abbreviated as BSB) (♂) (Qin et al. 2014a). 3nRR were derived from distant hybridization of the autotetraploid fish line and RCC, in which females can produce normal eggs, but males cannot produce sperm. Thus, these autotriploid fish provide a model system to study effects of triploidization on reproductive physiology in vertebrate. By studying the methylation levels and expression levels of meiosis-related genes in 3nCC and 3nRR, our data reveal that high expression of meiosis-related genes can contribute to restoration of bivalent pairing. This study extends the knowledge of the influence of polyploidy on meiosis of fish, and are also useful in clarifying aspects of fertility.

## Materials and Methods

### Sample Collection

Diploid *Carassius auratus* (2n = 100, abbreviated as 2nCC) were randomly selected from the Dongting Lake water system. 3nCC came from the self-crossing offspring of the male and female triploid *Carassius auratus* that were randomly selected from the Dongting Lake water system. Red crucian carp (2n = 100, abbreviated as RCC) and 3nRR were provided by the Engineering Center of Polyploid Fish Breeding of the National Education Ministry located at Hunan Normal University. All materials were obtained during breeding season (April) (2017–2018) and were cultured in open pools (0.067 ha) with suitable pH (7.0–8.5), water temperature (22–24°), dissolved oxygen content (5.0–8.0 mg/L), and adequate forage. Fish treatments were carried out according to the recommendations in the Guidelines for the Care and Use of Laboratory Animals of the National Advisory Committee for Laboratory Animal Research in China and approved by the Animal Care Committee of Hunan Normal University (Permit number: 4237). All samples were anesthetized with 100 mg/L MS-222 (Sigma-Aldrich, St Louis, MO, USA) before blood collection. Peripheral blood cells were extracted surgically.

### Fluorescence In Situ Hybridization

Chromosome preparations were performed using the peripheral blood cell cultures of all samples. The chromosomes were prepared in accordance with Qin et al. (2019). Species-specific centromere probes of fluorescence in situ hybridization (FISH) were made from RCC and amplified by PCR using the primers 5′-TTCGAAAAGAGAGAATAATCTA-3′ and 5′-AACTCGTCTAAACCCGAACTA-3′. Detailed steps were performed according to He et al. (2012).

### Measurement of DNA Content

To detect the DNA content of 3nCC blood and sperm, a flow cytometer (Partec GmbH) was employed. Detailed steps were performed according to Xiao et al. (2011).

### Gonadal Structure

Ten RCC, 10 2nCC, and 10 3nCC at age 9 months were randomly selected for histological observation of gonad structure, while 10 3nRR were randomly selected for observation of 21 months. Detailed steps were performed according to Qin et al. (2018).

### RNA Isolation and cDNA Synthesis

Extract RNA according to the instruction of Total RNA Kit I (Omega). The first-strand cDNA was synthesized using a PrimeScript™ RT reagent Kit with gDNA Eraser (Perfect Real Time, Takara). The obtained cDNA was stored at − 20 °C.

### CDS Region Cloning

The degenerate primers for *Ph1* and *Dmc1* were designed based on the nucleotide sequences found in zebrafish and other *Cyprinidae* fish (Table 1). According to the RT-PCR results, the target genes are mainly expressed in the gonads. Using the cDNA as template, their CDS regions were cloned from the gonad tissues. BioEdit and basic local alignment search tool (BLAST) were used to analyze the nucleotide and amino acid sequence alignments and perform the homology comparison.

**Table 1:** Primers used in cloning coding sequence, real-time qPCR, methyl-specific PCR

Primer name	Sequence
For cloning coding sequence	
*Ph1*-F	5′-ATGGAGGCAGGAGAAGACCAG-3′
*Ph1*-R	5′-TTAATCCCTTAGGCTGTTGATAG-3′
*Dmc1*-F	5′-ATGAAAACTTTAGAGGACCAGG-3′
*Dmc1*-R	5′-TTAGTCTTCGGCATCTGTTATT-3′
For real-time qPCR	
*Ph1*-qPCR-F	5′-CAACCGCTCCCAAACACCAAA-3′
*Ph1*-qPCR-R	5′-AAGGCGAGGAAGTCGGAGGA-3′
*Dmc1*-qPCR-F	5′-TCCACATCACAACAGGCAGTCTGGA-3′
*Dmc1*-qPCR-R	5′-CCAGGAAGCTGAGCGGTTACAC -3′
*Actin*-F	5′-GCCCTGCCCCATGCCATCCT-3′
*Actin*-R	5′-AGTGCCCATCTCCTGCTCGA-3′
For methyl-specific PCR	
*Ph1*-M-F	5′-GGAAGATTGTAGAGAAGATTAGAT-3′
*Ph1*-M-R	5′-ATCAATAATACTATAATTCAACCCT-3′
*Dmc1*-M-F	5′-TTGATGGTTTTGGTTATTTGAAAATA-3′
*Dmc1*-M-R	5′-AAAAATAAAACCACACCAACTTCTC-3′

### Quantitative Real-Time PCR Analysis

Quantitative real-time PCR (Prism 7500 Sequence Detection System, ABI) was used to study the expression of *Ph1* and *Dmc1* genes in 3nRR and 3nCC, using RCC and 2nCC as controls, and *β-actin* gene as an endogenous control (the primer described in Table 1), and for extraction of RNA from gonadal tissue for quantitative real-time PCR. Real-time qPCR-specific primers were designed based on identical sequences in the *Ph1* and *Dmc1* coding regions (Table 1). The results were analyzed according to the 2^−ΔΔCT^ method of Livark and Schmittgen (2001). Detailed steps were performed according to Duan et al. (2014).

### Methyl-Specific PCR

Using the zebrafish genome as a reference, the promoter regions of the *Ph1* and *Dmc1* genes were found using the Ensembl (http://asia.ensembl.org/index.html) website. Methylation primers were designed by predicting the promoter region CpG islands by the MethPrimer (http://www.urogene.org/cgi-bin/methprimer/methprimer.cgi) website (Table 1). Total genomic DNA was isolated from the gonads according to the Shanghai Sangon animal genomic DNA extraction kit. The extracted DNA was processed according to the instructions of the MethylCode™ Bisulfite Conversion Kit (Thermo Fisher). The clone was cloned and the cloned results were sent to Sangon for sequencing. Methylation of the resulting sequence is by BiQ analyzer.

## Results

### Fluorescence In Situ Hybridization and Measurement of DNA Content

**Fig. 1:**
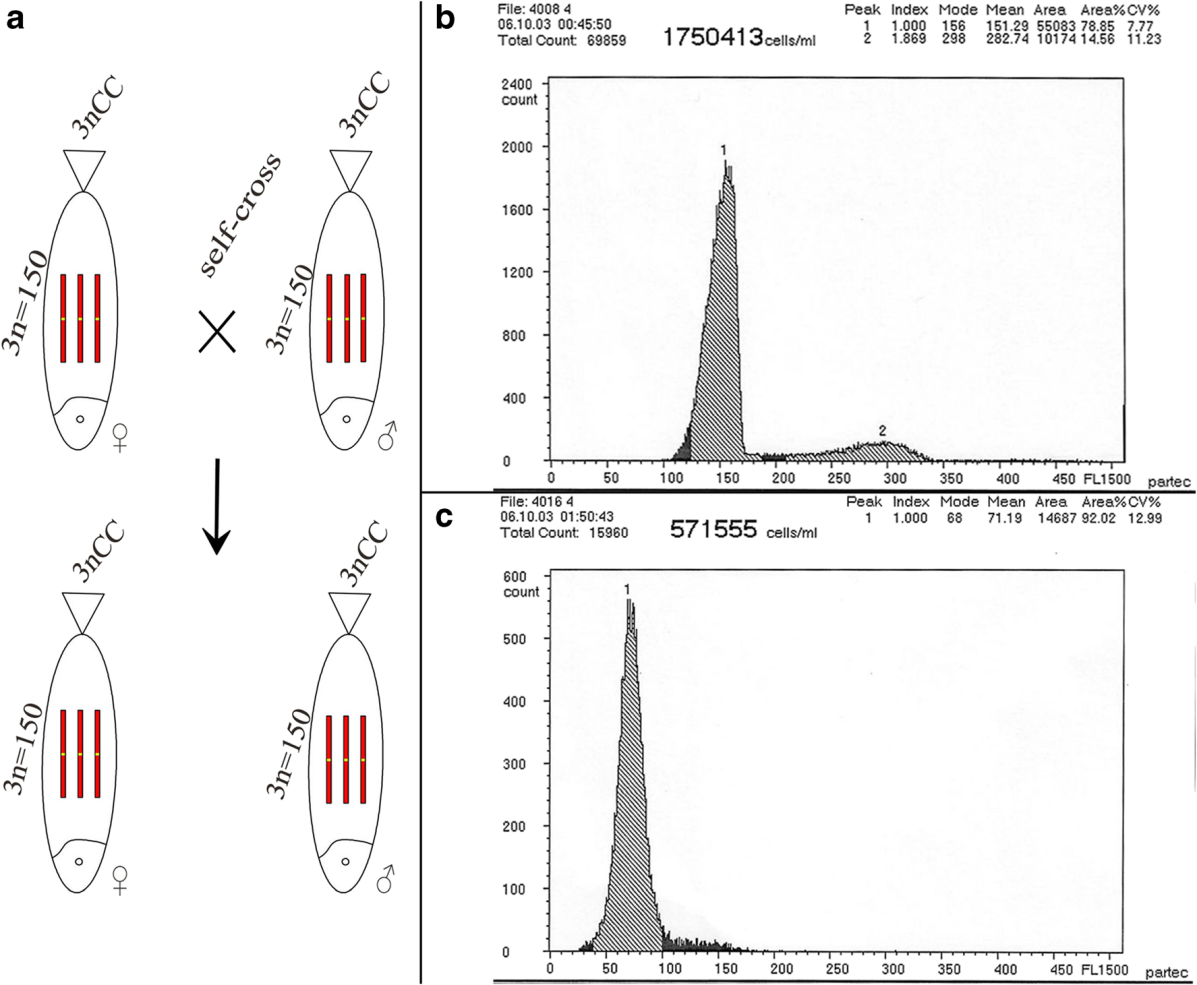
Formation of 3nCC. (A) The formation of 3nCC. (B) The mean DNA content of 3nCC. (C) The mean DNA content of 3nCC sperm. 3nCC, autotriploid *Carassius auratus*

3nCC produce bisexual autotriploid offspring when its ova were activated by 3nCC spermatozoa (Fig. 1a). We used the sum of the DNA content of 3nCC blood as the controls (Fig. 1b). The mean DNA content of 3nCC sperm was one-half to that of 3nCC blood (Fig. 1c), suggesting that 3nCC can produce reduced gamete.

**Fig. 2:**
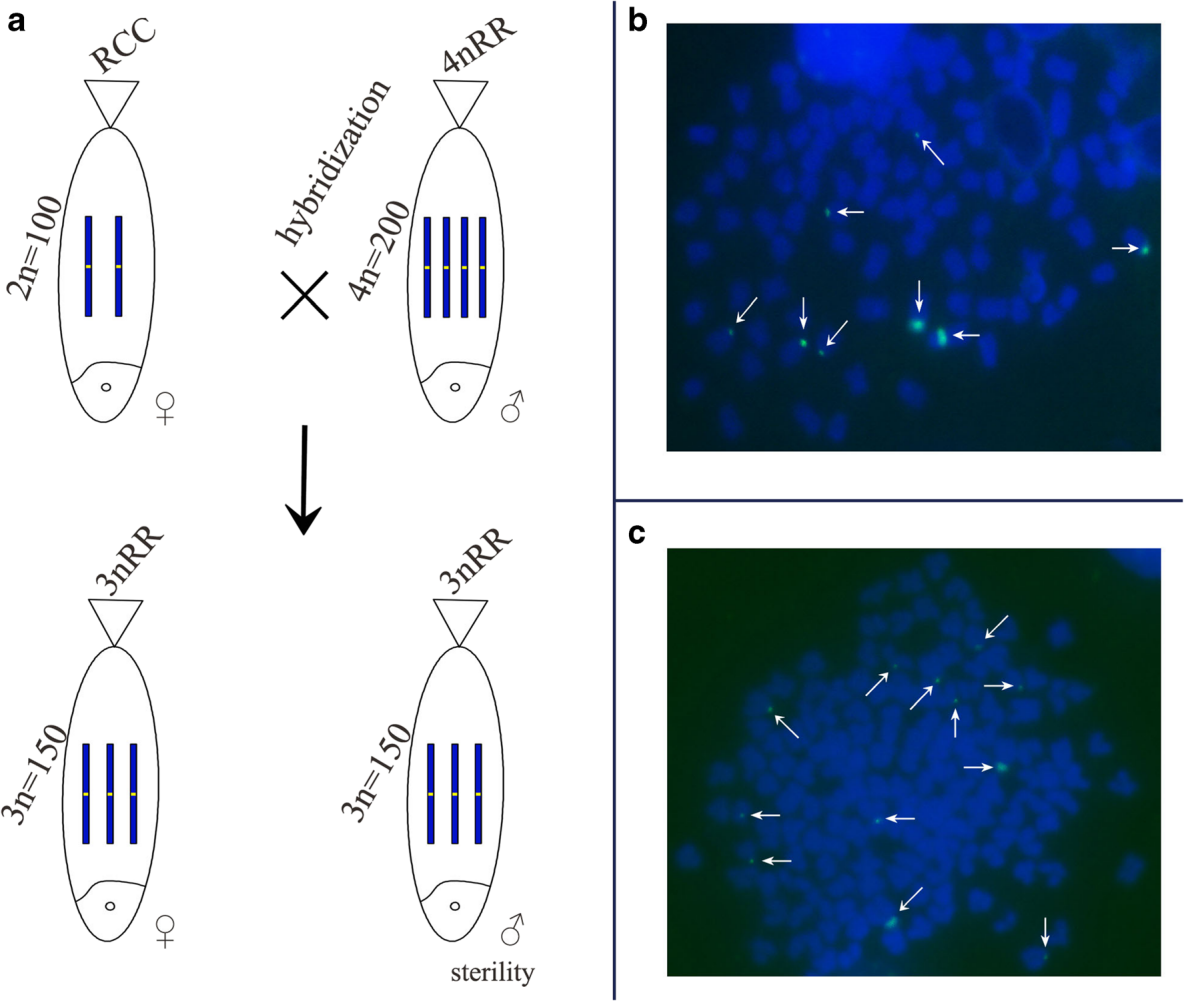
Formation of 3nRR. (A) The formation of 3nCC. (B), (C) Fish hybridisation signals in the metaphase chromosomes of RCC and 3nRR with class III (477 bp) 5S rDNA as a probe. The white arrows indicate the 5S rDNA gene loci. The eight 5S gene loci in RCC (A) and twelve in 3nRR (B). The bars in (A ,B) denote 3 μm. RCC, *Carassius auratus* red var.; 3nRR, autotriploid

3nRR were produced by distant hybridization of the autotetraploid fish line and RCC (Fig. 2a). The 5S gene probe (GenBank accession no. GQ485557) was hybridized to the metaphase chromosomes of RCC, BSB, 4nRR, and 3nRR, and the results of FISH are shown in Table 2. Hybridization of the probe yielded eight 5S gene loci in 91% of the chromosomal metaphases of RCC (Fig. 2b; Table 2). Twelve 5S rDNA loci were detected in 88% of the chromosomal metaphases of 3nRR (Fig. 2c; Table 2), suggesting that they were autotriploid and possessed three sets of RCC-derived chromosomes.

**Table 2:** Examination of hybridizing signals by FISH in RCC, BSB, 4nRR, and 3nRR

Fish type	No. of fish	No. of metaphase	No. of loci (477 bp)
RCC	10	200	8
BSB	10	200	0
4nRR	10	200	12
3nRR	10	200	16

### Gonadal Observations

**Fig. 3:**
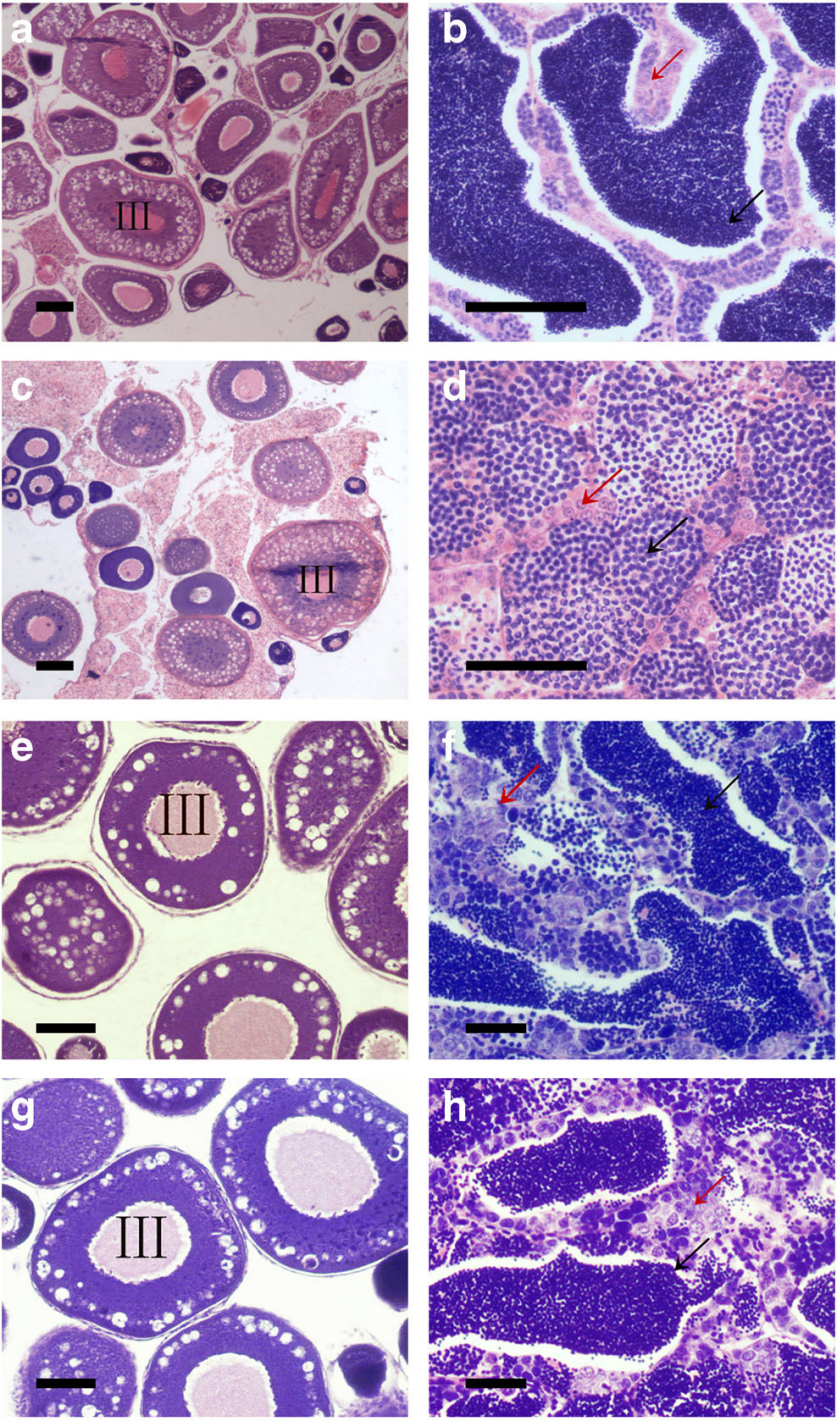
Gonad microstructure of the reproductive stage. (A) RCC ovary; (B) RCC testis; (C) 3nRR ovary; (D) 3nRR testis; (E) 2nCC ovary; (F) 2nCC testis; (G) 3nCC ovary; (H) 3nCC testis. In the picture, the red arrow points to the spermatocyte, and the black arrow points to the sperms. As the figure shows, 3nCC and 3nRR containing many phase III oocytes. The 3nCC testis contains a large number of mature sperm, while the sperm in the 3nRR testis are vacuolated or broken. Bar: 50 μm

Figure 3 shows the gonadal structure of RCC, 2nCC, 3nRR, and 3nCC at 10 month old. The ovaries of 3nCC (Fig. 3g) and 3nRR (Fig. 3c) contain many phase III oocytes, indicating that their ovaries can be fertile. The gonad structure of 3nCC (Fig. 3h) contains a large number of mature sperm, while the sperm of 3nRR (Fig. 3d) is vacuolated or broken. The results showed that 3nCC testes were fertile and 3nRR’s were not fertile.

### Cloning and Analysis of CDS Region

The complete CDS region sequences of *Ph1* and *Dmc1* genes of RCC, 3nRR, 2nCC, and 3nCC were cloned. The lengths of the coding regions of the *Ph1* gene of RCC, 3nRR, 2nCC, and 3nCC are 2664 bp, 2664 bp, 2649 bp, and 2649 bp, respectively. The *Ph1* gene CDS regions of RCC, 3nRR, 2nCC, and 3nCC encode proteins of 887, 887, 883, and 883 amino acids, respectively. The sequences had been deposited in GenBank (MH704442, MK140665, MH704443, and MH704873). The *Dmc1* gene coding regions of RCC, 3nRR, 2nCC, and 3nCC are all 1029 bp in length, encoding 342 amino acids. The sequences had been deposited in GenBank (MH973696, MK140666, MK140667, and MK140668). Sequence analysis using Clustal W (2.1) and DNAMAN revealed the existence of high homologies between 3nRR and 3nCC at the protein level, with amino acid identities of 92.3% for *Ph1*, 100% for *Dmc1* (Table 3). Nucleotide sequence alignment also revealed high homologies above 91.7%. Therefore, gene sequencing and homology analysis showed that the *Ph1* gene has significant homology at 3nRR and 3nCC, and the *Dmc1* gene is highly conserved.

**Table 3:** Percentage nucleotide identity (on the left) and percentage amino acid identity (on the right) between *Ph1* and *Dmc1* in RCC, 3nRR, 2nCC, and 3nCC

*Ph1*	RCC	3nRR	2nCC	3nCC
RCC	1/1	0.997/0.997	0.917/0.922	0.917/0.923
3nRR	0.997/0.997	1/1	0.917/0.922	0.917/0.923
2nCC	0.917/0.922	0.917/0.922	1/1	0.993/0.993
3nCC	0.917/0.923	0.917/0.923	0.993/0.993	1/1
*Dmc1*	RCC	3nRR	2nCC	3nCC
RCC	1/1	0.999/1	0.996/1	0.996/1
3nRR	0.999/1	1/1	0.995/1	0.995/1
2nCC	0.996/1	0.995/1	1/1	1/1
3nCC	0.996/1	0.995/1	1/1	1/1

### *Ph1* and *Dmc1* Gene Expression and Methyl-Specific PCR

**Fig. 4:**
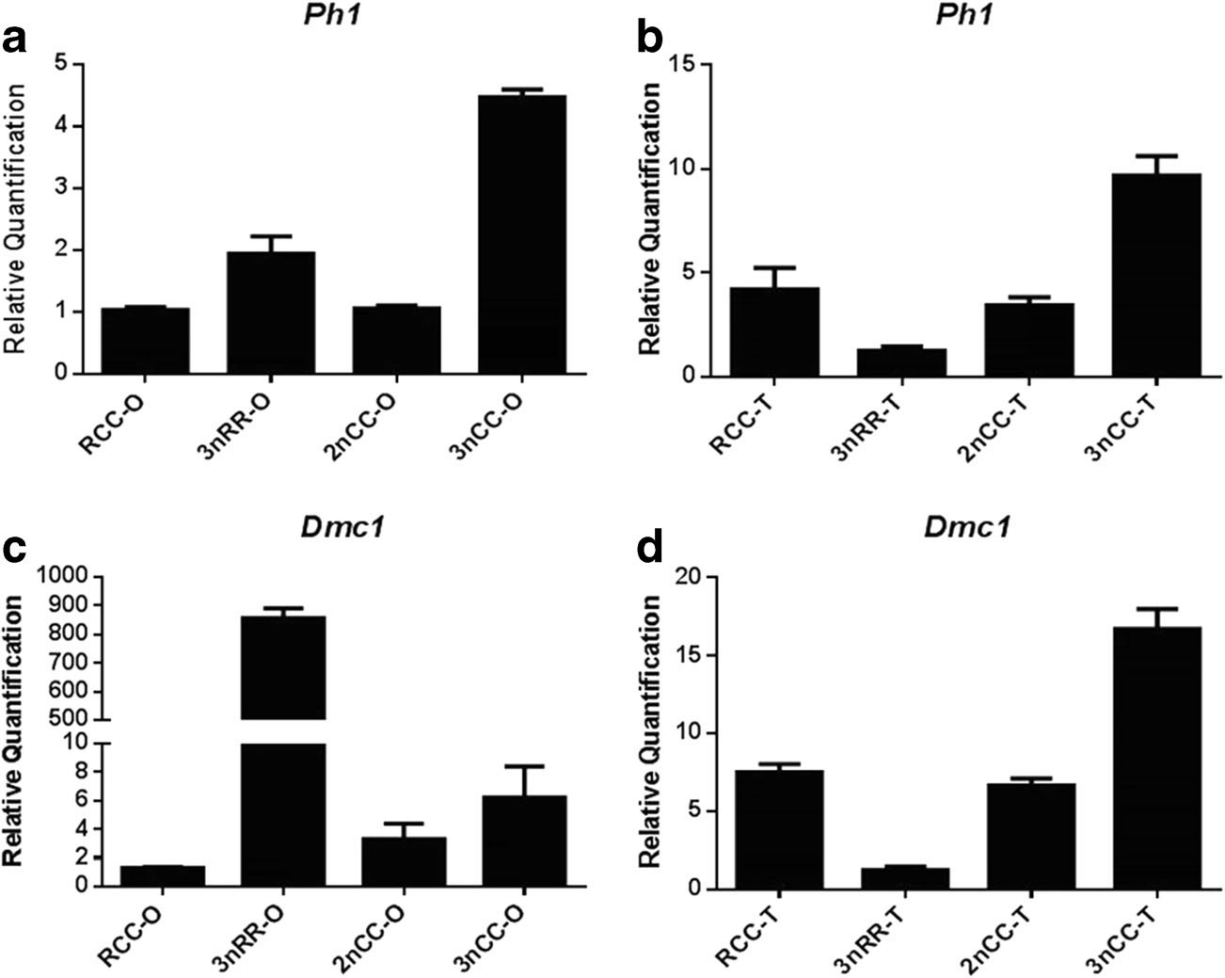
Relative expression of the *Ph1* and *Dmc1* genes in the gonads of RCC, 3nRR, 2nCC and 3nCC during the breeding season. Among them, (A) and (C) are the relative expression levels of *Ph1* and *Dmc1* in the ovary. (B) and (D) are the relative expression levels of *Ph1* and *Dmc1* in the testis

Comparison of expression levels of *Ph1* and *Dmc1* genes between 3nRR and 3nCC using quantitative real-time PCR using RCC and 2nCC as control (Fig. 4). The results showed that the *Ph1* and *Dmc1* genes were highly expressed in 3nCC male and female and 3nRR female individuals (*p* < 0.05) compared with 2nCC and RCC, but were lowly expressed in 3nRR male individuals (*p* < 0.05).

**Fig. 5:**
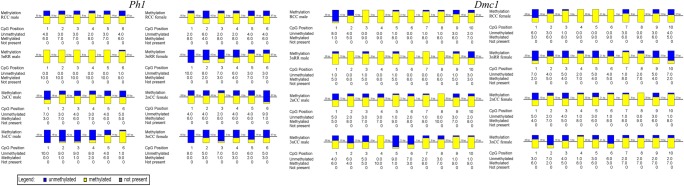
Sequencing results of methylation extent of the promoter regions of *Ph1* and *Dmc1* genes, wherein yellow represents methylation and blue represents no methylation

**Fig. 6:**
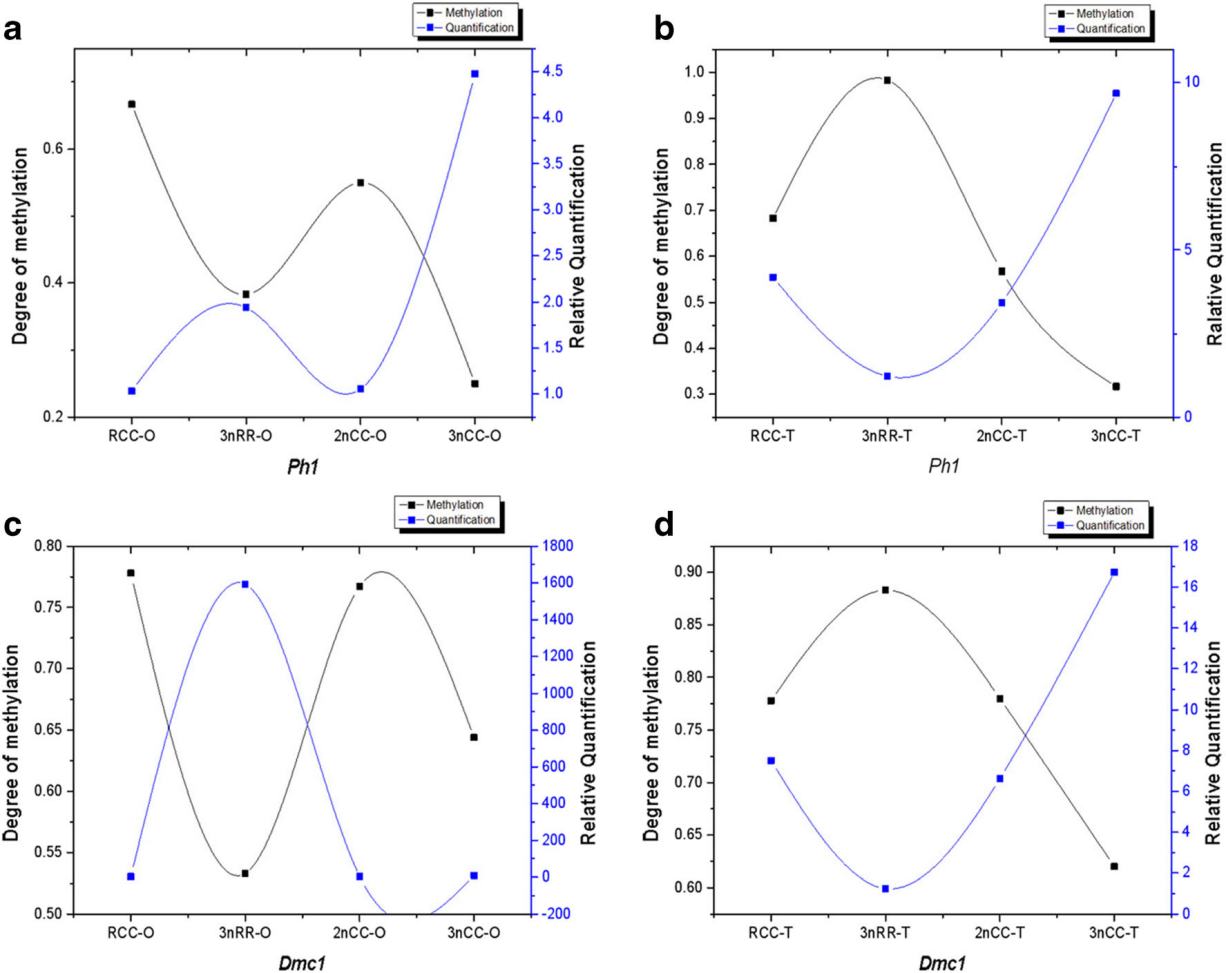
Correlation map of methylation level and expression level

The methylation status of the *Ph1* and *Dmc1* gene promoter regions of RCC, 3nRR, 2nCC, and 3nCC was shown in Table 4 and Fig. 5. The results showed that the methylation levels of the two gene promoter regions of 3nCC female individuals, male individuals, and 3nRR female individuals were lower than those of their diploid ancestors. The methylation levels of the promoter regions of *Ph1* and *Dmc1* in male individuals of 3nRR were 0.983 and 0.883 (Table 4), respectively, which were much higher than the methylation degree of their diploid ancestors and male 3nCC. There was a negative correlation between the methylation degree and the relative expression of promoter region of *Ph1* and *Dmc1* genes (Fig. 6).

**Table 4:** Methylation degree of *Ph1* and *Dmc1* promoter of RCC, 3nRR, 2nCC, and 3nCC

Name	Methylation degree
RCC-O-*Ph1*	0.667
RCC-T-*Ph1*	0.683
3nRR-O-*Ph1*	0.383
3nRR-T-*Ph1*	0.983
2nCC-O-*Ph1*	0.550
2nCC-T-*Ph1*	0.567
3nCC-O-*Ph1*	0.250
3nCC-T-*Ph1*	0.317
RCC-O-*Dmc1*	0.778
RCC-T-*Dmc1*	0.778
3nRR-O-*Dmc1*	0.533
3nRR-T-*Dmc1*	0.883
2nCC-O-*Dmc1*	0.767
2nCC-T-*Dmc1*	0.780
3nCC-O-*Dmc1*	0.644
3nCC-T*-Dmc1*	0.620

*O* ovary, *T* testis

## Discussion

Triploids are traditionally considered sterile (Ramsey and Schemske 2002; Cifuentes et al. 2013); however, recent research revealed that autotriploid fish are can be fertile and can produce normal gametes (Qin et al. 2015; Qin et al. 2016). The fertility of fish can be judged by observing gonad development during the breeding season, and the histological features of the gonads indicate that male and female 3nCC both have normal gonadal structures and can reach maturity at 1 year. Male 3nRR show a large number of vacuoles and broken sperm cells in the histological sections of the testis, while a normal ovarian structure is found in female individual. Our results indicate that 3nCC gonads can develop normally and produce gametes, and 3nRR can produce normal eggs without producing normal sperm. We conclude that autotriploidation does not strictly lead to the disappearance of fertility and does not lead to complete infertility.

*Ph1* and *Dmc1* are all important meiosis-related genes. The expression products of the former are necessary for meiotic synapse, and the latter is inducing specific binding of centromere. DMC1 deficiency leads to the abnormal formation of synaptic complex and the disordered separation of homologous chromosomes, and the deletion or mutation of *Dmc1* in human and mice can lead to spermatogenesis disorder and premature ovarian failure (Kagawa and Kurumizaka 2010; He et al. 2018). *Ph1* gene can promote homologous chromosome pairing, and it is also necessary for homologous chromosome pairing (Riley and Chapman 1958; Mellosampayo 1971). Previous studies have shown that various types of genomic changes are induced during the formation of polyploids (Chen and Pikaard 1997). In this study, the *Ph1* and *Dmc1* gene coding regions of 3nCC and 3nRR were 99% and 100% respectively compared with their diploid ancestors. It is concluded that the *Dmc1* and *Ph1* genes of 3nCC and 3nRR have no significant difference in nucleic acid sequence and can encode amino acids normally. These results suggest that autotriploid has no significant effect on the structure of *Ph1* and *Dmc1* genes.

Genome merging and doubling brings about plenty of gene redundancy (Soltis et al. 2004; Otto 2007). To overcome the unstable bottleneck caused by genomic doubling, a proper dose compensation mechanism must be used to regulate the appropriate amount of gene product (Pala et al. 2008). Complex dose compensation mechanisms induce a series of rapid genetic and epigenetic modifications (Bird et al. 2018). As a common epigenetic phenomenon, changes in DNA methylation can regulate gene expression (Costello et al. 1994; Futscher et al. 2002; Liu and Wendel 2003; Ma and Gustafson 2005; Koganti et al. 2017; Pang et al. 2015). DNA methylation is closely related to gametogenesis, embryonic development, sex differentiation, and sex determination (Li et al. 2019; Wei et al. 2018; Jiang et al. 2015; Gupta et al. 2012; Piferrer et al. 2012). In this study, compared with diploid ancestors, the *Ph1* and *Dmc1* genes are hypomethylated in all 3nCC individuals and female 3nRR individuals, while hypermethylated in male 3nRR individuals. The results of quantitative real-time PCR showed that *Ph1* and *Dmc1* genes were highly expressed in all 3nCC individuals and female 3nRR individuals, but a low expression in male 3nRR individuals. During the breeding season, 3nCC can extrude normal gametes and 3nRR can extrude eggs of different sizes without extruding semen. In meiosis, polyploids adopt a special mechanism of diploid meiosis to avoid the problem of gametes’ imbalance caused by complex chromosome combinations in metaphase I (Maestra et al. 2002). Two rounds of polyploidy origins in the gibel carp lead to the occurrence of the hexaploid gibel carp (Li et al. 2014). Thus, it is speculated that the 3nRR may be also hexaploid. Considering the fish-specific genome duplication that was regarded as having happened in the history of teleost evolution, crossbreeding leading to male sterility (Samuel et al. 2002; Taylor et al. 2003; Olivier et al. 2004; Vandepoele et al. 2004; Yoshikawa et al. 2018). We speculate that in the female individuals of 3nRR, due to the high expression of the *Ph1* and *Dmc1* genes, homologous chromosome pairing is promoted, resulting in the production of eggs of different sizes. Due to the high expression of the *Ph1* and *Dmc1* genes, the 3nCC has diploidized to some extent. The level of gene expression is regulated by the degree of methylation of the gene promoter region. In the data presented here, we cannot explain why the *Ph1* and *Dmc1* genes are hypermethylated in male 3nRR.

## Data Availability

The complete clean reads for these libraries have been uploaded to the NCBI. Sequence read archive site (http://www.ncbi.nlm.nih.gov/sra/; accession nos. MH704442, MK140665, MH704443, MH704873, MH973696, MK140666, MK140667, and MK140668).
